# Biologic therapies for Crohn’s disease: optimising the old and maximising the new

**DOI:** 10.12688/f1000research.18902.1

**Published:** 2019-07-29

**Authors:** Mark Samaan, Samantha Campbell, Georgina Cunningham, Aravind Gokul Tamilarasan, Peter M. Irving, Sara McCartney

**Affiliations:** 1 Department of Gastroenterology, University College London Hospitals NHS Foundation Trust, London, UK; 2 Department of Gastroenterology, Royal Free London NHS Foundation Trust, London, UK; 3 Department of Gastroenterology, Guy's and St Thomas' NHS Foundation Trust, London, UK

**Keywords:** Crohn’s disease, Biologics, Infliximab, Adalimumab, Vedolizumab, Ustekinumab

## Abstract

The era of biologic agents for the treatment of Crohn’s disease has brought about significant benefits for patients, and since the introduction of infliximab at the turn of the century, the entire field has moved on rapidly. Clinicians now have multiple agents at their disposal and a choice between several different anti-inflammatory mechanisms of action. This has allowed unprecedented improvements not only in symptoms and quality of life for patients previously refractory to conventional treatments but also for demonstrated healing of the intestinal mucosa and resolution of perianal fistulation. However, despite the undisputed efficacy of these agents, there remains a significant proportion of patients who fail to gain a meaningful benefit. Through years of studying infliximab and its counterpart anti-tumour necrosis factor (anti-TNF) agent, adalimumab, we now understand that strategies such as combining use with a conventional immunomodulator or measuring serum levels can help to optimise outcomes and reduce the proportion of patients for whom treatment fails. Work is ongoing to understand whether these principles apply to newer biologics such as vedolizumab and ustekinumab. In addition, novel approaches are being investigated in an attempt to maximise the benefit that these agents could offer. In this article, we summarise these new understandings and consider ways in which they could be integrated into clinical practice for the benefit of patients.

## Introduction

In the two decades since the advent of infliximab (IFX) for the treatment of Crohn’s disease (CD), biologic therapies have delivered substantial improvements in outcomes for patients with inflammatory bowel disease (IBD). Not only can they improve symptoms (resulting in demonstrably improved quality of life) but they also resolve inflammation, judged objectively using endoscopic, radiological or biochemical measures. In addition, biologic therapies have significantly changed the way in which perianal CD is managed and are currently our most effective pharmacological class of drugs for this particularly debilitating manifestation. The US Food and Drug Administration’s approval of IFX for CD in 1998 was followed 9 years later by the approval of adalimumab (ADA) and certolizumab (although IFX and ADA were subsequently approved in Europe, certolizumab was not). For several years, these agents with their common anti-tumour necrosis factor (anti-TNF) mechanism formed the entirety of the licensed biologic options available for CD. However, in recent years, the range of agents and mechanisms of action has expanded. First, in 2014, the selective leukocyte adhesion molecule inhibitor, vedolizumab (VDZ), was approved for use in ulcerative colitis (UC) as well as CD. This was followed in 2016 by the approval (for CD only) of ustekinumab (UST), a monoclonal antibody that targets the p40 subunit of interleukin-12 and interleukin-23. In addition to these new agents, the number of licensed treatments has been further expanded by the growing range of IFX and ADA biosimilar agents now available.

Whilst the large-scale registration trials by which the anti-TNF agents were granted their approvals clearly demonstrated their efficacy, it has taken many years and dedicated studies for us to gain a deeper understanding of how they should be used for maximum benefit. Examples include the benefit of their use in combination with a conventional immunosuppressant and introducing them earlier in the disease course as well as the use of therapeutic drug monitoring (TDM) for dose optimisation. Advances in our appreciation of these concepts have allowed practice to evolve to provide improved patient outcomes. Attention is now turning to investigating whether similar concepts will apply to newer biologic agents. In this review, we aim to draw together emerging data which provides new understanding of the optimal use of established agents and to consider how the impact of more novel biologics could potentially be optimised.

## Anti-tumour necrosis factor: old mechanism, new understandings

### Infliximab

***Dosing regimen modification.*** Standard dosing for IFX induction is a 5-mg/kg intravenous infusion at weeks 0, 2 and 6 and then every 8 weeks thereafter. However, there are a number of ways that this dosing regimen can be modified to optimise an individual’s therapy. In patients with low IFX trough levels (and absent or low-titre anti-drug antibodies) during maintenance therapy, intensifying IFX dosing can improve clinical outcomes and increase the number of patients achieving clinical response ^1^. This may be achieved either by increasing each infusion to 10 mg/kg or by shortening the dosing interval to either 4 or 6 weeks. Ideally, decisions regarding dose adjustment should be made with the benefit of TDM, inclusive of anti-drug antibody measurement. This is in view of the commonly encountered clinical scenarios for which dose intensification has less rationale. An example is active disease due to the development of high-titre antibodies with sub-therapeutic trough levels (immune-mediated pharmacokinetic failure) or adequate trough levels without antibodies (mechanistic/pharmacodynamic failure), which may warrant a change in therapy rather than dose intensification ^2^.

At the opposite end of the spectrum, patients in deep remission on IFX maintenance with supra-therapeutic trough levels could de-escalate their dosing, as relapse rates have been demonstrated to be low ^1, 3^. Again, this may be done by lengthening the inter-dose interval or reducing the concentration of the infusion (if previously receiving 10 mg/kg). The Trough Level Adapted Infliximab Treatment (TAXIT) study showed that dose reduction (targeting a trough level of 3 to 7 µg/mL) results in a similar proportion of patients in remission but with a 28% reduction in the associated drug costs ^1^.

Pregnancy presents another situation in which the dosing regimen of biologic agents may be modified. In this scenario, the aim of modification is usually to maintain the beneficial effect of treatment while attempting to limit exposure to the infant. It is known that both IFX and ADA can cross the placenta from the latter part of the second trimester ^4^. Therefore, in the setting of a sustained remission, many clinicians recommend temporary discontinuation from this point until after delivery. However, where there is evidence of ongoing disease activity or in the setting of previously refractory or complex disease, the risk-benefit often favours continuing treatment throughout. Although the mechanisms which allow IFX and ADA to cross the placenta are efficient enough to result in up to fourfold higher levels in infant and cord blood compared with maternal levels ^5, 6^, this does not appear to adversely affect the developing infant in the short term ^7^. Therefore, any putative benefit of reducing infant exposure should be balanced with the risks of an intra- or post-partum disease flare, of which there exists conflicting evidence. de Lima *et al*. reported no difference in relapse rates in women with sustained remission who stopped anti-TNF treatment before week 25 compared with those who continued therapy beyond week 30 (9.8% versus 15.6%, *P* = 0.14) ^8^. By contrast, a study by Groupe d’Etude Thérapeutique des Affections Inflammatoires Digestives (GETAID) observed a relatively high intra-partum (14%) or early post-partum (32%) relapse rate in mothers who discontinued therapy before week 30 ^9^. Where treatment is continued throughout pregnancy, recent evidence has shown that maternal IFX levels rise whilst ADA levels remain stable (after accounting for changes in albumin, body mass index and C-reactive protein [CRP]). It has therefore been suggested that TDM performed during the second trimester may help guide dosing during the third ^10^.

On balance, for patients who have disease requiring biologic therapy and who are contemplating pregnancy, ADA appears preferable to IFX ^11^. This is on the basis that rates of transportation of ADA across the placenta are lower ^12^ and that it is cleared more quickly from the circulation of infants than IFX ^7^. Nonetheless, it is important for clinicians and pregnant patients to be aware that European consensus recommends that live vaccines be avoided until after 6 months in infants exposed to anti-TNF *in utero* ^13^. There are clearly far less data guiding how best to manage more novel biologics, such as VDZ and UST, during pregnancy. Although both agents are expected to cross the placenta in a manner similar to that of IFX and ADA, there has so far been no evidence of harm ^14, 15^. However, further studies are required to better understand their safety and pharmacokinetics during this period.

***Measuring and monitoring.*** As is now widely appreciated, trough levels of IFX have been shown to correlate with clinical response, mucosal healing, and clinical remission. The TAXIT study established that targeting IFX trough levels to 3 to 7 µg/mL resulted in more efficient use of the drug ^1^. An analysis of trough-level thresholds showed a progressive reduction in the proportion of patients not achieving remission at lower levels. The rates decreased from 25% at a level of at least 1 µg/mL to 15% for those with a level of at least 3 µg/mL, 8% for levels of at least 5 µg/mL and 4% for at least 7 µg/mL. Taking into account these data in addition to several other studies, the American Gastroenterological Association (AGA) made a “conditional” recommendation that at least 5 µg/mL should be the target for IFX trough concentrations ^16^. However, it should be noted that TDM comprises just one aspect of monitoring in an attempt to maintain tight disease control and that dosing regimen modifications should be tailored to the individual. Moreover, although many clinicians advocate proactive TDM ^17^ (that is, dose adjustments based on TDM in asymptomatic patients), there currently exist little direct data to recommend this strategy. Indeed, the first published randomised trial (Study Investigating Tailored Treatment With Infliximab for Active Crohn's Disease, or TAILORIX) of proactive versus reactive (that is, in response to symptoms) dose adjustment strategies found no difference between the two ^18^.

One subset of patients that warrants separate discussion consists of the patients who have fistulating disease. It has been shown that higher IFX trough levels are associated with perianal fistula healing in both adults and children ^19, 20^. Yarur *et al*. performed a cross-sectional study showing that median IFX levels amongst patients with fistula healing were significantly higher than those without fistula healing (15.8 versus 4.4 µg/mL) ^20^. When levels were stratified by quartiles, a linear association between IFX trough levels and fistula healing was observed. In addition, the absence of anti-drug antibodies was shown to correlate with healing. The optimal levels for fistula healing were at least 10 µg/mL and some patients even required levels of at least 20 µg/mL ^20^. These findings suggest that trough levels that would usually be considered sufficient for the treatment of luminal disease may be inadequate to achieve fistula healing. Although current evidence does not suggest a relationship between drug exposure and adverse events ^21^, the safety of such high trough levels has not yet been confirmed in large or longitudinal cohorts.

***Combination with a conventional immunosuppressant.*** The immunogenicity of IFX and its clinical implications have been well established for some time. A recent review article analysing 114 studies reported that IFX immunogenicity rates ranged from 0 to 65.3% and were slightly higher for CD than UC ^22^. In addition, the proportions of patients achieving and maintaining a response were lower in those patients with detected anti-drug antibodies. Other outcomes, including adverse event data (for example, rates of infusion reactions) and trough IFX levels, were superior in those who did not develop anti-drug antibodies ^22^.

Robust data generated by the Study of Biologic and Immunomodulator Naive Patients in Crohn’s Disease (SONIC) trial demonstrated that combination therapy with azathioprine achieves higher remission rates in CD ^23^. Recent observational and randomised studies, in abstract form, have shown that combination therapy reduces the rates of immunogenicity. A large, prospective, observational UK-wide study from the Personalised Anti-TNF Therapy in Crohn’s disease (PANTS) investigator consortium showed immunogenicity rates for IFX (Remicade) of 26% at week 54 and 42% at 3 years (and similar results were seen for the IFX biosimilar CT-P13). These rates were reduced with immunomodulator use (hazard ratio (HR) = 0.37, *P* <0.0001) ^24^. Similarly, a recent randomised study showed that in those who failed ADA because of anti-drug antibody development, use of combination therapy (with azathioprine) when starting IFX significantly lowered the risk of immunogenicity ^25^.

Perhaps more compelling than short- and medium-term efficacy studies were the findings of the Randomized Evaluation of an Algorithm for Crohn’s Treatment (REACT) study, a cluster randomisation trial of treatment strategies ^26^. This demonstrated that early combined immunosuppression was associated with a reduced rate of major adverse outcomes (surgery, hospital admission or serious disease-related complications) compared with conventional management (27.7% and 35.1%, absolute difference 7.3%, HR 0.73, 95% confidence interval 0.62 to 0.86, *P* = 0.0003).

In summary, the high rates of IFX immunogenicity observed in the literature and the associated poorer clinical outcomes appear to offer support for the use of combination therapy wherever possible. More recently, a post-hoc analysis of the SONIC trial suggested that the benefit of adding azathioprine to IFX could be explained solely by the resulting increment in IFX serum concentrations rather than the additive immunosuppressive effect of azathioprine ^27^. In keeping with this, a subsequent prospective study observed that optimised IFX monotherapy is as effective as optimised combination therapy ^28^. However, the IFX monotherapy group required significantly higher rates of treatment escalation, which makes this strategy unfavourable on a cost-effectiveness basis. 

### Adalimumab

***Dosing regimen modification.*** Currently, the approved dosing schedule for ADA is to give 160 mg followed by 80 mg two weeks later and then a maintenance dose of 40 mg every two weeks. Unfortunately, there is still a proportion of patients who do not respond to or lose response to ADA. In patients who do not respond, increasing the dosing frequency to once a week has been shown to be effective in recapturing response in CD ^29^. However, to date, the data regarding dose escalation of ADA have been retrospective. To assess this prospectively, the Study to Evaluate Efficacy and Safety of Two Drug Regimens in Subjects With Moderate to Severe Crohn's Disease (SERENE-CD) trial has randomly assigned patients to receive higher induction and maintenance doses to establish whether primary and secondary loss of response can be avoided by maintaining higher serum drug concentrations from the outset ^30^. If a benefit is observed, this may lead to a change in the approved dosing regimen.

***Measuring and monitoring.*** Higher concentrations of serum drug are associated with better outcomes, not only clinical remission but also endoscopic healing and deeper histological remission ^31^. However, for ADA, unlike for IFX, there is evidence to suggest that it may not need to be a trough level that is taken. Ward *et al*. performed a prospective observational study on 19 patients with CD on maintenance ADA and took serum levels at multiple intervals during the usual 14-day cycle ^32^. From this, the authors concluded that although ideally a trough levels should be taken, if a level of at least 4.9 μg/mL is detected during the first 9 days following a dose, it can reasonably predict an adequate trough level ^32^. A recent retrospective study including 382 patients with IBD (311 of whom had CD) found that proactive TDM may be associated with a lower risk of ADA treatment failure compared with standard of care (defined here as either reactive TDM or empirical dose escalation) ^33^. However, a prospective randomised trial directly comparing these groups (to mirror the TAILORIX trial in IFX) has yet to be conducted.

For their 2017 guidelines, the AGA reviewed data from four studies that reported the proportion of patients not in remission above ADA trough concentrations of 5 ± 1 or 7.5 ± 1 µg/mL. This proportion progressively decreased from 17% at a threshold of 5 µg/mL to 10% at a threshold of 7.5 µg/mL. They therefore gave a “conditional” recommendation for the use of 7.5 µg/mL as the target trough concentration ^16^.

***Combination with a conventional immunosuppressant.*** There are varying results from real-world cohort studies in terms of the effect of concomitant immunomodulator therapy on response rates to ADA. Previously, it was felt that the addition of immunomodulators offered no additional benefit in terms of prevention of anti-drug antibodies ^34^. However, recent data from PANTS demonstrated that immunomodulators significantly reduce the immunogenicity of ADA (HR = 0.34, *P* <0.0001) ^24^.

## Newer mechanisms: tips and tricks

### Vedolizumab

**Figure 1.**
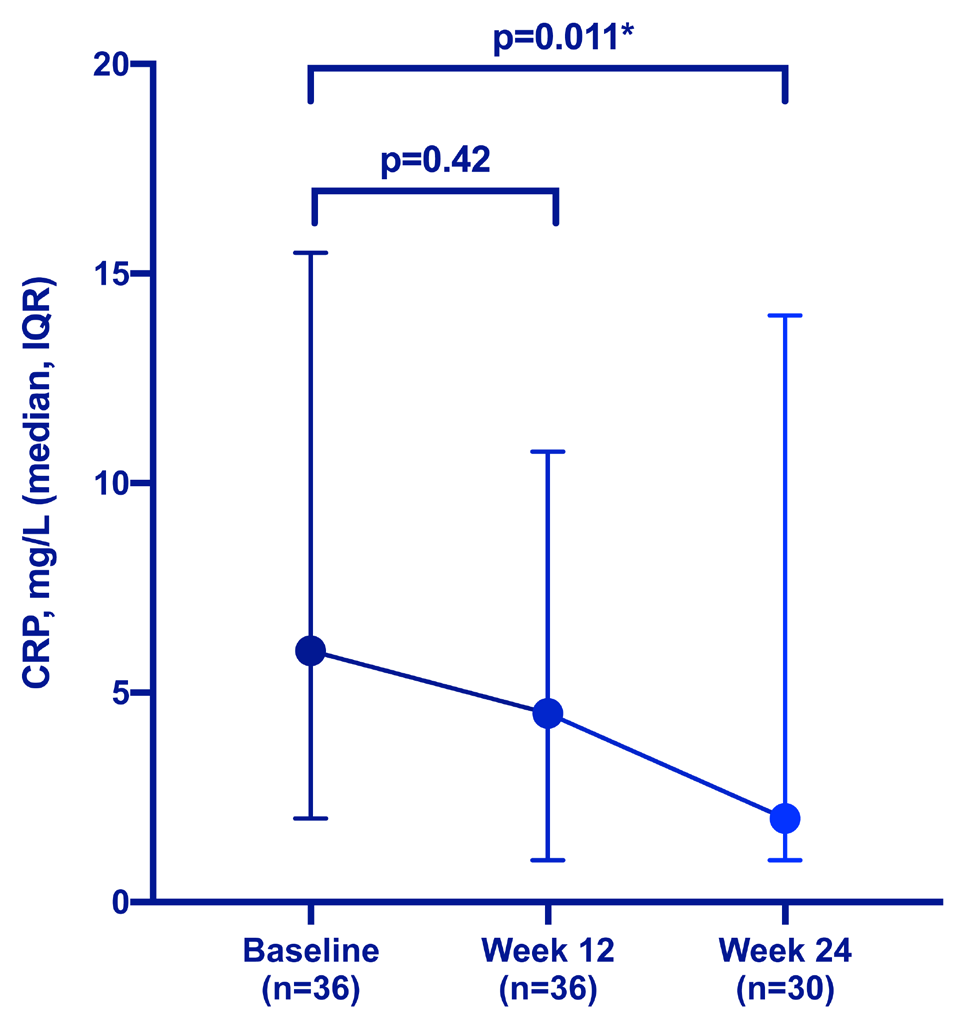
Change in C-reactive protein (CRP) following vedolizumab dose intensification. Vedolizumab dosing interval shortened from every 8 weeks to every 4 weeks among 36 inflammatory bowel disease patients with a previously suboptimal response. IQR, interquartile range. *denotes statistical significance ( *P* < 0.05).

***Dosing regimen modification.*** The induction and maintenance dosing schedule for VDZ involve an intravenous infusion at weeks 0, 2 and 6 and then every 8 weeks. The dose is standardised at 300 mg per infusion and is not weight-based like IFX. There is scope for variation in the maintenance dosing of VDZ to every 4 or 6 weeks, especially in those who are secondary non-responders. A 15% relapse rate was observed in one study that switched patients from every-4-week to every-8-week VDZ (and appears similar between UC and CD). Upon dose intensification back to every 4 weeks, 80% re-entered remission ^35^. This suggests that, in patients with CD, increasing dose frequency of VDZ to every 4 weeks could lead to an improvement. A recent meta-analysis echoed these findings of dose escalation to recapture response in secondary non-responders ^36^. The results revealed that a high proportion of patients with CD were secondary non-responders (47.9 per 100 patient-years) and that 56 (50%) out of 111 of secondary non-responders re-entered remission upon dose escalation ^36^. An observational cohort study of 36 patients with IBD (18 with UC and 18 with CD) with a previous suboptimal response to every-8-week dosing also demonstrated a significant reduction in CRP (from 6 to 2 mg/L, *P* = 0.011) after 24 weeks of every-4-week dosing ( Figure 1) ^37^. The reality of dose intensification with VDZ, although it is within licence, is that, in some healthcare systems, effectively doubling the cost of an already-high-cost drug is considered prohibitively expensive. The cost differential, in recent years, has become even more marked when comparing dose-intensified VDZ with biosimilar agents.

Analysis of patients in GEMINI II and III studies, who had an inadequate response at week 6 and received an additional infusion at week 10, suggested that this may improve chances of remission at week 52 ^38, 39^. Therefore, this optional addition to the standard induction regimen was included in the licence and should be integrated into clinical practice algorithms.

***Measuring and monitoring.*** A personalised treatment regimen with VDZ using a targeted therapeutic windows and drug levels requires further research. Unlike for anti-TNF agents, it is not yet a well-established tool used by clinicians as a guide for dose adjustment. Despite studies that have demonstrated that there may be potential value of TDM, the lack of unequivocal supporting data means that most changes in dose frequency are currently made empirically ^35^. To help guide response to induction and maintenance dosing, we recommend checking objective markers of disease activity at weeks 0 and 14. Ideally, this would include a clinical disease index score and faecal calprotectin and CRP measurements. In CD, additional monitoring at week 6 will help to determine the need for an extra dose at week 10.

A dose-response relationship for VDZ was observed in the GEMINI I, II and III studies. Drug level quartile analysis demonstrated a significantly higher rate of clinical response and remission, amongst patients the highest drug level quartile, compared to the lowest ^38– 40^. This observation was apparent during both the induction and maintenance phases of treatment. Subsequent literature suggests that week 6 drug level monitoring is a relevant predictor of remission or of the need for subsequent dose intensification ^41– 43^. One study reported that a week 6 level of over 20 µg/mL may be associated with improved clinical outcomes ^35^. Another observed an association between a week 6 level of over 18 µg/mL and increased rates of mucosal healing ^43^. The same study examined mucosal healing at week 52 compared with trough levels at weeks 2 and 14 (in addition to week 6), and only week 6 levels showed an association with mucosal healing ^43^. Drug levels and outcomes vary in the supporting literature and need further validation in larger prospective studies. Until data from larger studies are available, VDZ should be administered with dose optimisation based on objective measures of disease activity.

***Combination with a conventional immunosuppressant.*** In patients who receive VDZ, the development of antibodies appears to be low (<5%). This degree of immunogenicity appears unlikely to have a significant impact on clinical outcomes and may decrease over time ^38– 41, 44, 45^. GEMINI II and III studies reported immunogenicity rates of 4.1% and 1%, respectively. GEMINI II found persistent antibodies in just 0.4%, whereas GEMINI III found no persisting antibodies. Although these measurements were performed by using a drug-sensitive assay (and therefore were unable to detect antibodies in the presence of drug), the results have been broadly corroborated in other studies using drug-tolerant assays ^45^. Studies testing samples with drug-tolerant assays found low immunogenicity rates of 17% (7 of 41 patients) during the induction phase, 3% during the maintenance phase and 2.2% (4 of 179) after the first infusion ^44, 45^. When antibody development was demonstrated in the induction phase, 3 of the 7 responded to induction therapy ^45^. The transient phenomenon of immunogenicity using VDZ is supported by undetectable antibody burden by week 40 when detected after first infusion ^44^. The literature therefore supports low immunogenicity rates in treatment with VDZ and shows that antibody development is unlikely to have a significant impact on clinical outcomes.

Based on the majority of current evidence, combination therapy with immunomodulators to solely prevent immunogenicity does not appear to be a necessary strategy with VDZ. This is highlighted in a study that analysed pharmacokinetics and pharmacodynamics which demonstrated that the clearance and concentration of VDZ were not affected when co-administered with other immunomodulators ^46^. An integrated summary of VDZ suggests that whilst its low rates of immunogenicity could be further reduced by concomitant use of immunomodulators, the long-term risk-benefit should be evaluated and may not be in favour of their use ^47^. This risk-benefit analysis is clearly different from that of IFX, which has much higher rates of immunogenicity. The only note of caution in this regard is that there has been no dedicated randomised study comparing combination therapy with VDZ alone to provide a more definitive answer to this question.

VDZ appears to have a more gradual onset of action than other biologic agents, so initiation of treatment often requires bridging with other agents, at least until the end of induction. Corticosteroids are ideally placed to fulfil this role, but in those who are refractory, bridging with calcineurin inhibitors has been shown to be safe and effective ^48^. They can also aid in maintaining remission up to 52 weeks. In a study where calcineurin inhibitors were used alongside VDZ, 44% of patients with CD had achieved steroid-free remission at week 14 ^48^. This study had a small cohort and further studies are warranted.

### Ustekinumab

**Figure 2.**
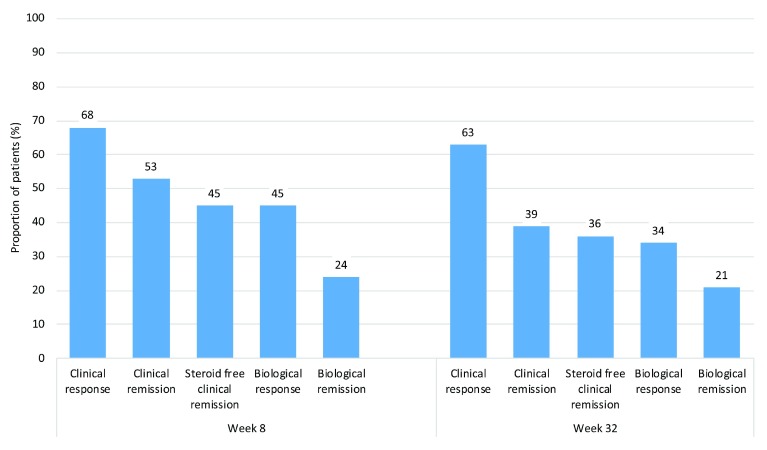
Clinical and biological outcomes at weeks 8 and 32 of 149 patients who received ustekinumab. Biological remission is defined as C-reactive protein (CRP) of less than 5 mg/L in patients with a baseline CRP of more than 5 mg/L. Biological response is defined as a 50% reduction in CRP. Remission is defined as a Harvey–Bradshaw index (HBI) score of less than 5 points. Response is defined as reduction in HBI score of at least 3 points or sustained HBI score of less than 5 points.

***Real-world effectiveness data.*** In addition to the randomised controlled trial data of ustekinumab’s efficacy demonstrated by the UNITI program, there exists a growing body of observational data of its effectiveness in clinical practice. One such study, carried out as a collaboration between three IBD centres in London, included a cohort of 149 patients and reported week 32 response and remission rates of 63% and 39%, respectively ^49^. These are broadly consistent with another large cohort (n = 167) from Canada which described corresponding week 24 rates of 60% and 25% ^50^. The London cohort also reported a range of other endpoints, including biological response (50% reduction in CRP) and biological remission (CRP of less than 5 mg/L in patients with a baseline CRP of more than 5 mg/L) ( Figure 2) ^49^.

***Dosing regimen modification.*** The induction dosing schedule for UST is fixed and involves an intravenous infusion of 6 mg/kg at week 0 followed by a subcutaneous 90-mg dose at week 8. There is scope for variation in the frequency of maintenance dosing. Depending on response, subsequent 90-mg subcutaneous maintenance doses are given every 8 or 12 weeks. The maintenance dosing regimen of UST, unlike that of other biologics, is guided by an assessment of response to induction therapy. This requirement encourages good clinical practice. However, it can pose certain difficulties and requires additional investigations.

To establish adequacy of response to UST induction, a review at or just prior to week 16 is advised. Different modalities—including paired Harvey–Bradshaw index (HBI) scores and biochemical markers such as CRP and faecal calprotectin, endoscopic or radiological evaluation—may be used to determine treatment effect. However, the feasibility and acceptability of repeating endoscopic or radiological evaluations within this time frame are likely to limit their utility in this context. Nonetheless, the well-recognised discrepancy between clinical and endoscopic remission in CD should be borne in mind. For example, a recent prospective study of UST induction showed an endoscopic remission rate at week 24 of 7.1%. The concurrent clinical remission rate was 39.5% ^51^. In summary, given the feasibility issues at play as well as the desire to use an objective marker in combination with symptoms ^52^, faecal calprotectin appears to be ideally placed ^53^. However, this can be applied only to patients with an elevated measurement at baseline.

If an adequate response is achieved, every-12-week dosing is considered appropriate, but in cases of partial response, every-8-week dosing is recommended. In circumstances where there is a deterioration or complete non-response, switching to another treatment (or surgery) is appropriate. However, it is recognised that in some patients a late response is seen, and given that patients may have already failed other therapies, it would be reasonable to persist and suggest a further every-8-week dose after week 16 ^54^. Another factor for consideration is that rates of endoscopic response and healing appear to be more favourable in every-8-week rather than every-12-week dosing ^55^. Therefore, if there is any doubt as to which dosing regimen to use, literature supports a decision to opt for every-8-week dosing.

There is also scope to adjust the frequency of maintenance dosing, even once already established. Post-hoc analysis of randomised controlled trials and observational evidence support changing from every-12-week to every-8-week dosing if a loss of response is observed ^54, 56, 57^. Dosing frequency can be reduced from every 8 weeks to every 12 weeks if remission is sustained, although there are little data to show the effect of this.

Another important practical factor for clinical use was addressed by a retrospective Canadian study that examined the rates of peri-operative complications associated with the use of UST (20 patients) compared with anti-TNF therapy (40 patients). Their findings suggested that there was no difference between the two groups with a post-surgical follow-up of 6 months, although the UST cohort of patients were more likely to be on concomitant immunomodulatory therapy and were more often undergoing surgery as an emergency. These findings will be reassuring to clinicians but should be confirmed in prospective studies with a larger cohort of patients ^58^.

***Measuring and monitoring.*** Access to UST TDM is currently limited and therefore it has not yet been integrated into routine clinical practice. However, a dose-response relationship has been demonstrated, and measuring serum levels will most likely be a useful tool to guide maintenance dosing in the future ^51, 56, 59^. For example, one recent study showed higher UST levels in endoscopic responders at every study time point (weeks 4, 8, 16 and 24) ^51^. In addition, potential therapeutic thresholds have already been postulated. A post-hoc analysis of UNITI suggested that a maintenance serum trough level of UST in the range of 0.8 to 1.4 µg/mL predicted clinical remission at 6 months ^56^. However, for endoscopic remission, higher levels appear to be required. A recent study suggested that a minimum UST drug level of 1.9 µg/mL was necessary to achieve an endoscopic response at 6 months ^51^. Another study suggested that a maintenance trough concentration of more than 4.5 µg/mL at 26 weeks correlated with biomarker and endoscopic response ^59^. However, there exists some discrepancy between these observations; in the former study, only 1.6% of patients had a 26-week trough level over 4.5 µg/mL. This may reflect insufficient dosing even whilst on an every-8-week regimen ^51^. It is possible that, in some cases of non-response, intensification of the dosing regimen (outside of licence) from every 8 weeks to every 4 weeks provides more adequate drug exposure and increased remission rates. However, further research in this regard is needed. The variation in serum levels between studies could be explained by intra-assay variation and other factors such as high baseline albumin, lower baseline faecal calprotectin and female sex (which are shown to be independent predictors of higher levels during induction ^51^). Currently, UST level concentration assays are not commercially available on a wide scale, so despite the growing body of evidence, at present dose adjustments are made empirically.

***Combination with a conventional immunosuppressant.*** In patients who receive UST, immunogenicity rates appear to be low. In addition, the use of concomitant immunomodulatory therapy does not seem to affect serum concentrations. Both of these findings were observed in a comprehensive pharmacokinetic and pharmacodynamic analysis of the UNITI trials ^56^. A post-hoc analysis of the UNITI trial revealed anti-UST antibodies in just 2.3% of 1366 patients during a year of treatment. These low rates are mirrored in another study showing antibodies in roughly 2% of patients (1 of 57) at weeks 8 and 16 ^51, 56^. The former study used a drug-tolerant assay, the latter a drug-sensitive assay. Although the use of concomitant immunomodulation does not appear to impact UST concentrations ^56^, prior exposure to anti-TNF agents and amount of previous anti-TNF exposure have been shown to negatively influence maintenance serum UST levels ^51^. It may be appropriate to consider discontinuation of immunomodulatory therapies after commencing UST if the sole reason for their use was to reduce immunogenicity.

## Unanswered questions and controversies

Despite great strides in terms of understanding how best to use biologic agents, there remain many unanswered questions. For example, even though no clear evidence suggests that using UST or VDZ in combination with an immunosuppressant is beneficial, it must be remembered that this did not become apparent for IFX until a dedicated trial, specifically aiming to answer that question, had been conducted. This trial is yet to be conducted for UST or VDZ. We also have a relatively limited understanding of their efficacy for IBD manifestations such as perianal fistulation and pouchitis, and most of the data for these indications come from post-hoc analyses of randomised trials and observational studies. In addition, although there have been no signals of harm, we currently have a relatively limited understanding of their safety in pregnancy, a factor which can play a significant role in guiding treatment decisions.

An ongoing source of debate and controversy in the era of multiple biologic mechanisms is biologic sequencing. We now have to decide not only which mechanism—and, in the case of anti-TNF, which agent—to use first but also which mechanism to switch to in cases of treatment failure. Although it is can be clearly appreciated from their registration trials that VDZ and UST are less efficacious when used after anti-TNF, it is not yet clear whether the efficacy of the anti-TNF agents is diminished but prior exposure to novel biologics. There is also interest in the concept of combining biologic agents with differing and perhaps complementary mechanisms of action. For example, a trial is under way which combines VDZ, ADA and methotrexate for CD patients considered at high risk for complicated disease ^60^. Although biologic combination regimens currently appear unfeasible on a cost basis alone, the impact of biosimilar versions is likely to make this type of strategy more achievable in the future. Finally, we have some way to go in terms of predicting which patients would benefit most from biologic therapy. In an attempt to address this question, the currently recruiting Predicting Outcomes for Crohn’s Disease Using a Molecular Biomarker (PROFILE) study uses a biomarker panel to separate CD patients at diagnosis into two cohorts: those likely to develop severe disease and those predicted to have a milder disease course. Patients within each group will be randomly assigned to receive either combination therapy with an immunomodulator and IFX (“top down” treatment) from the point of diagnosis or a “step up” approach of an immunomodulator initially followed by IFX only in the case of refractory disease. The trialists predict that patients in the severe group will benefit more from biologic treatment from the point of diagnosis, and if this is proven correct, it is likely that biologic therapies will appear earlier in treatment algorithms for this group of patients.

One thing that appears certain is that the range of biologic (and small-molecule) agents available for the treatment of CD will continue to expand—and rapidly. With several novel agents in late-phase trials and many more in earlier phases, the degree of complexity when making treatment choices will increase. Most importantly, with it, so will the range of options we have to offer patients with CD.

## Conclusions

Biologic therapies have completely changed the landscape of IBD care, and although there is renewed interest in small-molecule therapies, they appear likely to remain central to the management of patients with more severe or refractory disease. Despite this, there remains a great unmet need with many patients failing to respond to induction therapy or losing response after an initial improvement. Only through a broad spectrum of work carried out over many years were we able to fully appreciate the nuances necessary to optimise the effect of the anti-TNF agents. Indeed, we are still learning. Although some of this understanding can be extrapolated to help us maximise the benefit of novel biologics, such as VDZ and UST, an entirely new program of work is necessary. The fact that much of this research is already under way is cause for optimism and means that over time we will hopefully be able to meet the unmet need of patients with IBD.
